# Artificial intelligence readiness and innovative work behavior among Chinese university teachers: the indirect role of work engagement and the moderating role of perceived digital leadership

**DOI:** 10.3389/fpsyg.2026.1958619

**Published:** 2026-09-09

**Authors:** Li Fu, Jianbing Yan

**Affiliations:** School of Marxism, Zhengzhou Tourism College, Henan, China

**Keywords:** artificial intelligence readiness, innovative work behavior, job demands-resources theory, perceived digital leadership, work engagement

## Abstract

**Introduction:**

Artificial intelligence (AI) is increasingly being integrated into university teaching, research, and administration. However, it remains unclear whether and how AI readiness, as an AI-specific personal resource, is associated with broader innovative work behavior beyond AI adoption or explicitly AI-enhanced innovation. Drawing on Job Demands-Resources theory, this study examined the relationships among AI readiness, work engagement, and innovative work behavior, together with the moderating role of perceived digital leadership.

**Methods:**

Cross-sectional survey data were collected from 432 university teachers in China. AI readiness and work engagement were modeled as reflective-reflective higher-order constructs, whereas perceived digital leadership and innovative work behavior were specified as first-order reflective constructs. The hypothesized relationships were tested using partial least squares structural equation modeling.

**Results:**

AI readiness was positively associated with both work engagement and innovative work behavior, while work engagement was positively associated with innovative work behavior. The statistical indirect effect of AI readiness on innovative work behavior through work engagement was significant, with the direct association remaining significant. Perceived digital leadership strengthened the positive association between AI readiness and work engagement, although the interaction effect was very small.

**Discussion:**

The findings are consistent with a resource-conversion account in which AI readiness may have cross-domain relevance for broader workplace innovation, with work engagement representing a plausible motivational pathway through which this resource is activated. Perceived digital leadership may provide a complementary enabling condition, but its limited effect warrants cautious interpretation. Given the cross-sectional, self-reported data, the results indicate statistical associations rather than causal relationships.

## 1 Introduction

Artificial intelligence (AI) is increasingly embedded in higher education, supporting teaching, assessment, student services, research, and related academic tasks. Reviews of AI in education show a rapid expansion of applications but also a persistent imbalance: much of the literature has concentrated on student-facing systems and technical applications, with less attention to teachers and the organizational conditions surrounding their use (Celik et al., 2022; Crompton and Burke, 2023; Tan et al., 2025). The emergence of generative AI has widened the range of academic tasks that can be supported, but university teachers' responses remain mixed. A systematic review by Nikolic et al. (2024) found that teaching academics recognized potential gains in efficiency and instructional practice while also expressing concerns about reliability, academic integrity, skill development, training, and institutional policy. Lee et al. (2024) similarly documented uncertainty among educators regarding appropriate use and institutional preparedness. Taken together, this evidence suggests that access to AI does not by itself produce meaningful changes in academic work: technology availability cannot explain why some teachers translate AI into innovative practice whereas others do not.

An outcome that captures such change is innovative work behavior (IWB), which involves the intentional generation, promotion, and implementation of new ideas in a work role (Scott and Bruce, 1994; Janssen, 2000; De Jong and Den Hartog, 2010). Unlike technology adoption, which concerns whether a particular system is accepted or used, IWB concerns whether employees actively alter how work is performed and persist in bringing ideas into practice (Anderson et al., 2014). For university teachers, this may involve redesigning courses and assessments, experimenting with research procedures, improving student support, or developing more effective academic and administrative processes. Educational innovation therefore extends beyond idea generation to obtaining support and sustaining implementation (Lambriex-Schmitz et al., 2020). Evidence outside higher education also suggests that employees' capacity to sense opportunities and critically evaluate generative AI is associated with innovative work behavior, whereas technical use alone may be insufficient (Held and Heubeck, 2025). IWB is therefore an appropriate outcome for examining whether AI-related preparedness is associated with changes in teachers' professional practice, including innovations that are not themselves explicitly AI based.

AI readiness offers one possible explanation. It refers to teachers' preparedness to understand, evaluate, and use AI responsibly in their professional activities. Wang et al. (2023) conceptualized teachers' AI readiness as a multidimensional construct reflected in cognition, ability, vision, and ethics. The construct is related to, but not interchangeable with, adjacent concepts. AI literacy emphasizes the knowledge and competencies required to understand, use, and critically evaluate AI (Ng et al., 2021; Sperling et al., 2024). Technology acceptance focuses primarily on beliefs and intentions concerning a particular technology (Davis, 1989), while technology readiness refers to a more general propensity to embrace new technologies (Parasuraman, 2000). Organizational AI readiness, in turn, concerns firm-level strategic, technological, and organizational conditions (Jöhnk et al., 2020). Teachers' AI readiness instead captures whether individual educators possess the AI-related understanding, practical capability, foresight, and ethical judgment required for professional action. Although Wang et al. (2023) found that AI readiness was positively associated with AI-enhanced innovation among primary-school teachers, it remains uncertain whether overall AI readiness is associated with the broader innovative work behavior of university teachers across teaching, research, academic service, and administration.

However, the unresolved theoretical question is not simply whether AI readiness is associated with innovation, but whether and how an AI-specific form of preparedness is translated into innovative work behavior beyond AI adoption or explicitly AI-enhanced innovation. This represents both a domain-transfer and a resource-conversion question: whether a technology-specific personal capacity can support innovation across a broader range of professional activities, and how the possession of such capacity is motivationally mobilized into innovative action.

Job Demands-Resources (JD-R) theory provides a basis for explaining how this association may arise. The theory proposes that job and personal resources facilitate goal attainment, adaptation, and sustained motivation (Bakker and Demerouti, 2017). Personal resources are positively related to work engagement because they strengthen employees' perceived capacity to manage demands and influence their work environment (Xanthopoulou et al., 2007). AI readiness is not a conventional global personal resource such as optimism or resilience; it is treated here as a technology-specific resource with resource-like functions because it may increase teachers' perceived control over AI-related tasks, facilitate professional goal attainment, and support adaptation to technological change. Research among teachers has shown that job resources are associated with engagement and can become particularly important when work demands are high (Hakanen et al., 2006; Bakker et al., 2007). Engagement, in turn, provides the energy and persistence required to develop, promote, and implement new practices, and previous studies have connected engagement with personal initiative and workplace innovation (Hakanen et al., 2008; Kwon and Kim, 2020). Although previous studies have established a positive association between work engagement and innovative work behavior, they do not explain whether engagement represents the motivational state through which a multidimensional, AI-specific capacity is mobilized toward broader innovative behavior. Examining this indirect association therefore helps distinguish the possession of AI-related capacity from its motivational activation.

The motivational value of AI readiness may also depend on the leadership context in which it is exercised. In this study, perceived digital leadership (PDL) refers to teachers' perceptions that their immediate academic leader understands digital technologies, communicates a positive direction for digital transformation, and supports the structures and processes required for change. This individual-level formulation is consistent with research that measures employees' perceptions of leaders' digital competence and transformation-oriented behavior (Zeike et al., 2019). It also differs from an objectively shared university-level digital leadership climate. Leadership can shape the availability and usability of job resources and the conditions under which employees mobilize personal capabilities (Tummers and Bakker, 2021). Digital leadership research in higher education likewise emphasizes vision, coordination, capability development, and organizational support, while noting that theoretically grounded empirical evidence remains limited (Jameson et al., 2022). Related organizational evidence indicates that perceived digital leadership can be associated with employee work engagement (Li et al., 2024). AI readiness may therefore supply the personal capacity to engage with AI, whereas PDL may provide direction, legitimacy, and encouragement for exercising that capacity. Stronger PDL should make AI readiness more motivationally valuable; when PDL is weak, teachers' preparedness may remain an underused capability.

China provides a relevant setting for examining these relationships because universities occupy a prominent position in the country's AI-development and educational-digitalization strategies. The Artificial Intelligence Innovation Action Plan for Institutions of Higher Education called on universities to strengthen AI research, talent development, curriculum provision, and the translation of technological advances into education (Ministry of Education of the People's Republic of China, 2018). More recent national initiatives have continued to promote educational digitalization, teachers' digital capabilities, and the integration of AI into educational development (Ministry of Education of the People's Republic of China, 2025). Research has begun to examine AI adoption and organizational performance in Chinese higher education institutions (Mai, 2025), while evidence from Chinese university teachers has connected work engagement with innovative work behavior (Zhou et al., 2025). Nevertheless, national policy direction and institutional technology access do not explain how individual teachers respond to AI-related change or whether their preparedness is translated into innovative work behavior. Examining teachers' AI readiness together with their perceptions of immediate academic leaders may therefore provide a more differentiated account of innovation during policy-driven digital transformation.

Drawing on JD-R theory, this study examines whether AI readiness is associated with innovative work behavior among university teachers in China, whether work engagement constitutes a statistical indirect pathway in this relationship, and whether perceived digital leadership moderates the association between AI readiness and work engagement. These relationships are examined using cross-sectional survey data from 432 university teachers. This study develops a resource-conversion account of AI-enabled academic work rather than merely applying an established organizational behavior model to a new occupational context. First, it conceptualizes AI readiness as a domain-specific personal resource and examines whether its relevance extends beyond AI adoption or explicitly AI-enhanced innovation to broader innovative work behavior across teaching, research, academic service, and administration. This extends the theoretical boundary of AI readiness from technology-specific application to cross-domain professional innovation. Second, the study distinguishes resource possession from motivational activation. AI readiness reflects whether teachers possess relevant AI-related knowledge, ability, foresight, and ethical judgment, whereas work engagement reflects the energy and involvement invested in mobilizing these capacities. The statistical indirect association through work engagement therefore provides evidence consistent with a capacity-motivation-behavior account of innovation during AI-related change. Third, perceived digital leadership is examined as a contextual boundary condition that may influence whether teachers perceive the use of their AI-related capacities as supported and worthwhile. Because perceived digital leadership is broader than AI-specific leadership, this boundary-condition argument is interpreted cautiously. Together, these contributions refine the JD-R motivational perspective by explaining how an emerging technology-specific resource may be associated with broader innovative work behavior.

## 2 Literature review and hypothesis development

### 2.1 Theoretical foundation: job demands-resources theory

The Job Demands-Resources (JD-R) theory provides the principal theoretical foundation for this study. The theory distinguishes job demands, which require sustained physical or psychological effort, from job resources, which facilitate goal attainment, reduce the costs associated with demands, and support learning and development (Demerouti et al., 2001; Bakker and Demerouti, 2017). Subsequent developments have emphasized the flexibility of the framework across occupations and the reciprocal relationships that may develop among resources, engagement, and work outcomes (Bakker et al., 2023). JD-R theory proposes two related processes. In the health-impairment process, excessive job demands deplete employees' energy and may produce strain. In the motivational process, job and personal resources promote work engagement and, in turn, favorable work-related outcomes. Because the present study focuses on teachers' preparedness, engagement, and innovative behavior, it draws primarily on the motivational process.

Importantly, the present model does not include an explicit measure of job demands and should therefore not be interpreted as a test of the complete JD-R dual-process framework, the health-impairment process, or demand-resource buffering. Instead, JD-R theory is used as a focused explanatory lens for the motivational process. Its particular value for the present model lies in distinguishing the possession of a domain-specific personal resource (AI readiness) from its motivational activation (work engagement) and subsequent behavioral expression (innovative work behavior), while also recognizing that a contextual resource may facilitate this conversion. AI-related demands are theoretically acknowledged but remain outside the empirical model.

Personal resources generally refer to positive beliefs and person-held capacities that help individuals exercise control over their work environment and pursue valued goals (Xanthopoulou et al., 2007). They can function together with job resources and may develop reciprocal relationships with work engagement over time (Xanthopoulou et al., 2009). AI readiness is not equivalent to conventional personal resources such as optimism, resilience, or generalized self-efficacy. It is treated here as a technology-specific resource with resource-like functions because it may increase teachers' perceived control over AI-related tasks, facilitate professional goal attainment, and support adaptation to technological change. Teachers who understand AI, can use and evaluate it, anticipate its implications, and make responsible decisions about its application should be better equipped to manage the opportunities and uncertainties associated with AI-enabled academic work (Wang et al., 2023).

AI readiness is conceptualized as comprising cognition, ability, vision, and ethics (Wang et al., 2023). Cognition concerns teachers' understanding of AI and its educational implications; ability concerns the capacity to apply and evaluate AI tools; vision reflects the ability to anticipate how AI may reshape education and professional roles; and ethics concerns responsible judgment about privacy, bias, transparency, intellectual property, and accountability. These components overlap with aspects of AI literacy, particularly critical evaluation and ethical understanding, but AI readiness additionally concerns preparedness to mobilize such capabilities in professional action (Ng et al., 2021; Sperling et al., 2024). In specifying AI readiness as a reflective-reflective higher-order construct, this study assumes that the four dimensions covary as manifestations of teachers' broader preparedness to engage with AI. This specification does not imply that the dimensions are conceptually identical or that alternative formative representations are invalid.

The unresolved theoretical issue is not whether personal resources are generally associated with work engagement or whether engagement is associated with innovative behavior, as these relationships are already well established. Rather, the issue concerns whether a resource developed in relation to a specific technological transformation can have behavioral relevance beyond that technological domain. AI readiness is anchored specifically in teachers' preparedness for AI, whereas innovative work behavior encompasses the generation, promotion, and implementation of new practices across teaching, research, academic service, and administration. Examining their relationship therefore addresses a domain-transfer question: whether an AI-specific personal resource can support broader professional innovation rather than only AI adoption or explicitly AI-enhanced innovation.

Work engagement represents the motivational state through which AI readiness may become relevant to teachers' work behavior. It comprises vigor, dedication, and absorption, which are modeled as related manifestations of an overarching state of engagement (Schaufeli et al., 2002, 2006). Teacher-focused JD-R research has shown that resources are positively associated with engagement and can buffer the motivational consequences of demanding work conditions (Hakanen et al., 2006; Bakker et al., 2007). AI readiness may make technology-related work more manageable and meaningful, but possessing AI-related capacity does not necessarily mean that teachers will invest this capacity in innovative action. Work engagement is therefore conceptualized as the motivational activation of AI readiness rather than simply as another favorable employee outcome. AI readiness represents whether teachers possess relevant AI-related capacity, work engagement represents the energy and involvement invested in mobilizing that capacity, and innovative work behavior represents its potential behavioral expression. This capacity–motivation–behavior distinction constitutes the resource-conversion process examined in the present study.

Leadership can influence how employees mobilize their personal resources. A systematic review of leadership within JD-R theory suggests that leaders may shape job demands and resources and alter the extent to which available resources are translated into employee outcomes (Tummers and Bakker, 2021). Digital and e-leadership research likewise emphasizes leaders' role in articulating direction, coordinating technology-enabled change, and creating supportive conditions for employees (Avolio et al., 2014; Jameson et al., 2022). In this study, perceived digital leadership refers specifically to teachers' perceptions of their immediate academic leaders' digital knowledge, orientation toward digital technologies, and efforts to advance digital transformation (Zeike et al., 2019). It is therefore an individual-level perception of leader behavior, not an objective department- or university-level digital climate.

Taken together, the proposed framework refines the JD-R motivational process in two related ways. First, it extends the concept of personal resources from conventional domain-general resources, such as optimism and generalized self-efficacy, to a domain-specific form of preparedness associated with an emerging technological transformation. The model examines whether this AI-specific resource is associated with innovative behavior beyond explicitly AI-based activities, thereby testing its potential cross-domain relevance. Second, the framework distinguishes resource possession from resource mobilization. AI readiness represents teachers' AI-related capacity, whereas work engagement represents the motivational investment through which this capacity may become associated with broader innovative behavior. The direct relationship between AI readiness and innovative work behavior is additionally supported by a task-capability argument, while the indirect relationship through work engagement represents the proposed resource-conversion pathway. Perceived digital leadership is incorporated as a contextual boundary condition that may provide direction, legitimacy, and encouragement for mobilizing AI-related capacity. It is treated as a broad digital context rather than as an AI-specific leadership construct. Thus, the theoretical contribution lies not in proposing previously unexamined bivariate relationships, but in integrating domain transfer, motivational activation, and contextual resource complementarity within AI-enabled academic work.

### 2.2 AI readiness and university teachers' innovative work behavior

Innovative work behavior refers to employees' intentional efforts to identify problems and opportunities, generate and promote ideas, and implement those ideas in their work roles or organizations (Scott and Bruce, 1994; Janssen, 2000; De Jong and Den Hartog, 2010). It is broader than creativity, which is principally concerned with producing novel and useful ideas. Innovative work behavior also involves communicating an idea's value, securing support, overcoming resistance, and translating it into practice (Anderson et al., 2014). Among university teachers, it may include redesigning course content, developing new assessment methods, experimenting with pedagogical approaches, improving research procedures, or revising academic and administrative practices (Hosseini and Haghighi Shirazi, 2021; Lambriex-Schmitz et al., 2020).

AI readiness refers to teachers' preparedness to understand, evaluate, and use AI responsibly in their professional work. Its four dimensions may support different aspects of innovation. Cognitive readiness may help teachers identify tasks or problems for which AI offers a useful solution. Ability readiness enables them to evaluate outputs and convert ideas into workable applications. Vision readiness encourages consideration of how AI may alter established academic practices and create longer-term opportunities. Ethical readiness supports the evaluation and implementation of innovations within acceptable professional and institutional boundaries. Research on AI literacy similarly emphasizes that effective use requires critical evaluation and ethical understanding in addition to operational skill (Ng et al., 2021; Sperling et al., 2024).

The relevance of AI readiness is not necessarily limited to explicitly AI-based innovation. Engagement with AI can change how teachers recognize, represent, and address work-related problems. AI-related knowledge may expand the range of solutions considered, evaluative capability may improve comparisons among alternative ideas, and practical capability may reduce the time and effort required to test new approaches. These benefits may support innovation in teaching, research, academic service, and administration even when the resulting practice is not defined solely by its use of AI. AI readiness is therefore conceptually distinct from technology acceptance, which focuses on beliefs and intentions regarding a system (Davis, 1989), and from general technology readiness, which concerns a broader propensity to embrace new technologies (Parasuraman, 2000). It also differs from organizational AI readiness, which concerns firm-level strategic, technological, and organizational conditions (Jöhnk et al., 2020).

Available evidence provides initial support for the proposed relationship. Wang et al. (2023), drawing on data from 3,164 teachers, found that cognition, ability, vision, and ethics were positively associated with AI-enhanced educational innovation. Research on teachers' digital competence has also linked technology-related capabilities with innovative work behavior, although these constructs are not equivalent to AI readiness (Astuti and Setiawan, 2023). In a broader employment context, Held and Heubeck (2025) reported that employees' capacity to evaluate generative AI was positively associated with innovative work behavior, whereas the ability to operate AI tools alone did not show the same direct relationship. This distinction suggests that innovation depends not simply on technical use but also on employees' capacity to interpret AI outputs and apply them to meaningful work problems.

Nevertheless, existing studies have focused mainly on school teachers, explicitly AI-enhanced innovation, general digital competence, or employees outside higher education. University teachers perform a wider combination of teaching, research, academic service, and administrative responsibilities. It therefore remains unclear whether an AI-specific resource has cross-domain relevance for broader innovative work behavior. Accordingly:

**H1. AI readiness is positively related to university teachers' innovative work behavior**.

### 2.3 AI readiness and work engagement

Work engagement is a positive and fulfilling work-related state characterized by vigor, dedication, and absorption (Schaufeli et al., 2002, 2006). Vigor refers to energy and persistence at work, dedication reflects enthusiasm, significance, and commitment, and absorption denotes sustained concentration in work activities. Within JD-R theory, personal resources are expected to support engagement because they strengthen employees' perceived capacity to manage work demands and make the investment of effort appear more worthwhile (Xanthopoulou et al., 2007; Bakker and Demerouti, 2017). Evidence among teachers has similarly linked job resources with stronger work engagement (Hakanen et al., 2006).

The growing use of AI creates both opportunities and demands for university teachers. AI may support lesson preparation, assessment, feedback, information analysis, research, and routine administrative work. At the same time, its use requires teachers to acquire new competencies, evaluate rapidly changing tools, and adjust established professional routines (Celik et al., 2022; Lee et al., 2024). Technology-related change may also generate uncertainty concerning professional identity, job design, and the future allocation of work (Brougham and Haar, 2017), while intensive technology use may produce technostress when demands exceed the resources available to address them (Tarafdar et al., 2014).

The relationship between AI readiness and work engagement is therefore not theoretically unambiguous. Greater AI readiness may make AI-related learning demands, professional uncertainty, and ethical risks more salient. Teachers with stronger cognitive and ethical readiness may be more likely to recognize problems concerning privacy, algorithmic bias, academic integrity, authorship, intellectual property, and accountability. Similarly, stronger vision readiness may heighten awareness of potential changes in professional roles and future job design. Reviews of educators' responses to AI consistently identify reliability, integrity, fairness, training, and institutional guidance as material concerns (Celik et al., 2022; Nikolic et al., 2024). Such awareness may increase caution, perceived burden, or strain and could consequently weaken work engagement. AI readiness should therefore not be equated with uncritical enthusiasm for AI; it may operate through both an enabling mechanism and a burden-salience mechanism.

Nevertheless, the enabling mechanism is expected to predominate at the level of the overall AI-readiness construct because AI readiness involves not only awareness of AI-related challenges but also the capacity to evaluate and respond to them. Cognitive readiness may reduce ambiguity by helping teachers understand what AI can and cannot do. Ability readiness may lower the effort required to evaluate and apply AI tools. Vision readiness may facilitate purposeful adaptation to technological change, while ethical readiness may provide clearer criteria for distinguishing responsible applications from inappropriate ones. In JD-R terms, these resource-like functions may strengthen teachers' perceived control, help them manage AI-related demands, and make the investment of effort more meaningful and worthwhile. The proposed positive relationship therefore represents an expected net association rather than an assumption that every dimension of AI readiness will increase engagement under all conditions.

Direct evidence connecting AI readiness with work engagement remains limited. However, related research supports the proposed resource mechanism. Studies based on JD-R theory have shown that job and personal resources are positively associated with teachers' engagement (Hakanen et al., 2006; Xanthopoulou et al., 2007), while longitudinal evidence indicates that personal resources and work engagement may reinforce one another over time (Xanthopoulou et al., 2009). Technology readiness has been positively associated with work engagement among working adults (Khoza et al., 2024), and research on digitally mediated work indicates that job resources and positive technology-related experiences can support engagement (Moreira-Fontán et al., 2019; Rick et al., 2024). In higher education, Sang et al. (2023) found that digital competence was positively associated with Chinese university instructors' work engagement, while Fan et al. (2023) reported that digital information-literacy self-efficacy was positively related to the vigor, dedication, and absorption of Chinese university teachers.

These studies concern constructs adjacent to, rather than equivalent to, AI readiness. Nevertheless, they suggest that technology-related capacities may support engagement by making digitally mediated work more understandable, manageable, and purposeful. On balance, although AI readiness may heighten awareness of AI-related demands and risks, its combined cognitive, practical, visionary, and ethical capacities are expected to strengthen teachers' ability to respond to these challenges and remain engaged in their work. Accordingly:

**H2. AI readiness is positively related to university teachers' work engagement**.

### 2.4 Work engagement and university teachers' innovative work behavior

Innovative work behavior generally requires effort beyond the routine fulfillment of formal job responsibilities. Generating an idea is only an initial step. Employees must often refine the idea, communicate its value, obtain support, respond to resistance, and sustain implementation despite uncertain outcomes (Janssen, 2000; De Jong and Den Hartog, 2010). Daily evidence also indicates that engaged employees are more likely to display innovative behavior when they have autonomy and confidence in their creative capabilities (Orth and Volmer, 2017). For teachers, these activities require energy, persistence, concentration, and continued psychological investment.

The three dimensions of work engagement are closely aligned with these requirements. Vigor provides the energy and persistence needed to explore problems and respond to obstacles. Dedication gives teachers a sense of purpose and may increase their willingness to question ineffective routines. Absorption supports the sustained attention required to develop, test, and refine new practices. Work engagement represents a motivational state, whereas innovative work behavior refers to discretionary actions directed toward the generation, promotion, and implementation of ideas. Engaged teachers should therefore be more likely to invest effort in innovation rather than limit their behavior to prescribed duties (Hakanen et al., 2008; Kwon and Kim, 2020).

Accumulated evidence supports this relationship. Studies across organizational settings have reported positive associations between work engagement and innovative work behavior (Agarwal, 2014; De Spiegelaere et al., 2014; Orth and Volmer, 2017), and Kwon and Kim's (2020) integrative review of 34 empirical studies reached the same general conclusion. Evidence from higher education is also developing. Dixit and Upadhyay (2021) linked faculty engagement with innovative work behavior within a JD-R framework, while Hassan et al. (2024) found that engagement was positively related to academic staff innovation. Owusu et al. (2025) reported comparable evidence among teachers, and Zhou et al. (2025) identified a positive relationship among university teachers in China. These findings indicate that work engagement is not only an indicator of occupational wellbeing but also an important motivational correlate of teacher innovation. Thus:

**H3. Work engagement is positively related to university teachers**' **innovative work behavior**.

### 2.5 The indirect role of work engagement

AI readiness provides teachers with the capacity to engage productively with AI-related change, but possessing such capacity does not necessarily result in innovative action. Teachers may understand AI and be capable of using it while remaining unwilling to invest the effort required to depart from established routines. JD-R theory identifies engagement as a motivational state through which resources can become associated with proactive and discretionary work outcomes (Bakker and Demerouti, 2017; Kwon and Kim, 2020). Work engagement may therefore provide the motivational connection through which teachers mobilize their AI-related knowledge and capabilities in developing, promoting, and implementing new practices.

Previous studies have identified work engagement as an intervening variable connecting personal or contextual resources with innovative behavior. Work engagement has transmitted associations involving social-exchange relationships and job resources (Agarwal, 2014; De Spiegelaere et al., 2014), cultural intelligence (Afsar et al., 2020), and proactive or supportive work conditions (Dixit and Upadhyay, 2021; Nguyen and Petchsawang, 2024). Research involving academic staff has similarly shown that decent or supportive work conditions are associated with innovative behavior through engagement (Hassan et al., 2024), while Zhou et al. (2025) identified an indirect role of work engagement among Chinese university teachers. These studies do not examine AI readiness as the focal antecedent, leaving the proposed indirect association insufficiently understood.

Because the data in the present study were obtained in a single survey, the proposed ordering is theoretically derived rather than temporally demonstrated. The analysis therefore evaluates whether AI readiness has a positive statistical indirect association with innovative work behavior through work engagement, rather than establishing a causal process. Accordingly:

**H4. AI readiness is positively and indirectly associated with university teachers' innovative work behavior through work engagement**.

### 2.6 The moderating role of perceived digital leadership

Perceived digital leadership (PDL) refers in this study to teachers' perceptions that their immediate academic leaders possess current digital knowledge, maintain a positive orientation toward digital technologies, proactively advance digital transformation, and encourage others to participate in that transformation (Zeike et al., 2019). This operationalization is related to the broader e-leadership literature, which examines leadership processes mediated by advanced information technologies (Avolio et al., 2014), and to recent reviews emphasizing digital vision, capability development, and change coordination (Lin, 2024). PDL is treated here as an individual-level perception of immediate leader behavior rather than as an objective characteristic of the university or a shared organizational-level digital climate.

AI readiness and PDL operate at different levels of conceptual specificity. AI readiness is an AI-specific personal resource reflecting teachers' cognition, practical ability, vision, and ethical judgment concerning AI. PDL, by contrast, concerns leaders' orientation toward digital technologies and digital transformation more broadly. Digitalization encompasses a wide range of technologies, processes, and organizational changes, whereas AI represents one specific technological domain within this broader transformation. Accordingly, AI and digitalization are related but not equivalent concepts, and the PDL measure used in this study should not be interpreted as a measure of AI-specific leadership.

Despite this difference in specificity, broader digital leadership may still influence whether teachers mobilize their AI-related capacities. The usefulness of a personal resource depends partly on whether employees perceive opportunities and legitimacy to exercise it. Teachers may possess strong AI readiness but hesitate to invest that capacity in their work when the direction of digital change is unclear, experimentation appears inconsistent with departmental priorities, or technology-enabled initiatives receive limited support. Leaders can shape the availability and usability of resources and signal which forms of discretionary effort are legitimate and valued (Tummers and Bakker, 2021). Teachers who perceive their immediate leaders as knowledgeable about digital change and supportive of technology-enabled experimentation may therefore be more likely to regard the responsible application of their AI-related capabilities as worthwhile and consistent with unit priorities.

The proposed interaction can consequently be understood as resource complementarity across different levels of content specificity. AI readiness supplies the personal capacity to understand, evaluate, and use AI responsibly, whereas PDL may provide a broader contextual basis of direction, legitimacy, coordination, and encouragement for exercising that capacity. PDL does not create or replace teachers' AI readiness. Instead, it may increase the perceived opportunity and organizational acceptability of investing AI-related capacities in professional activities. This reasoning is consistent with the JD-R proposition that leadership can influence the availability and usability of resources and alter the strength of resource–outcome relationships (Tummers and Bakker, 2021).

Nevertheless, the correspondence between AI readiness and PDL is partial rather than exact. General digital leadership does not necessarily provide AI-specific strategy, specialized training, access to approved AI tools, or guidance concerning privacy, algorithmic bias, intellectual property, transparency, and accountability. An AI-specific leadership construct addressing these issues would therefore provide greater content congruence and theoretical proximity to AI readiness. The present study examines whether a broader digital leadership context has directional relevance for the motivational use of AI readiness; it does not assume that general PDL is equivalent to AI-specific leadership or that it represents the most proximal contextual moderator. The difference in content specificity may also constrain the magnitude of the proposed interaction.

Available empirical evidence is relevant but not conclusive. Alajmi (2022) found that school principals' digital leadership was positively associated with teachers' technology integration in Kuwait, although that study used a different instrument and examined school-level technology integration rather than AI readiness. Jameson et al. (2022) emphasized the importance of leadership in coordinating digital transformation in higher education but concluded that the field still lacked sufficiently mature and theoretically grounded evidence. Beyond education, digital leadership has been associated with work engagement and innovative work behavior (Ahmed et al., 2024; Li et al., 2024). Related evidence has also connected middle managers' perceived digital leadership with employee work engagement (Li et al., 2024). However, these studies concern digital change broadly and do not directly demonstrate that PDL strengthens the relationship between AI readiness and work engagement.

Taken together, general PDL may strengthen the AI readiness-engagement relationship by providing direction, legitimacy, and encouragement for technology-enabled experimentation. This proposed moderation reflects the complementarity between an AI-specific personal resource and a broader contextual resource, rather than an assumption that AI and digitalization are conceptually equivalent. Accordingly:

**H5. Perceived digital leadership positively moderates the relationship between AI readiness and work engagement, such that the relationship is stronger when perceived digital leadership is higher**.

### 2.7 Conceptual framework

**Figure 1:**
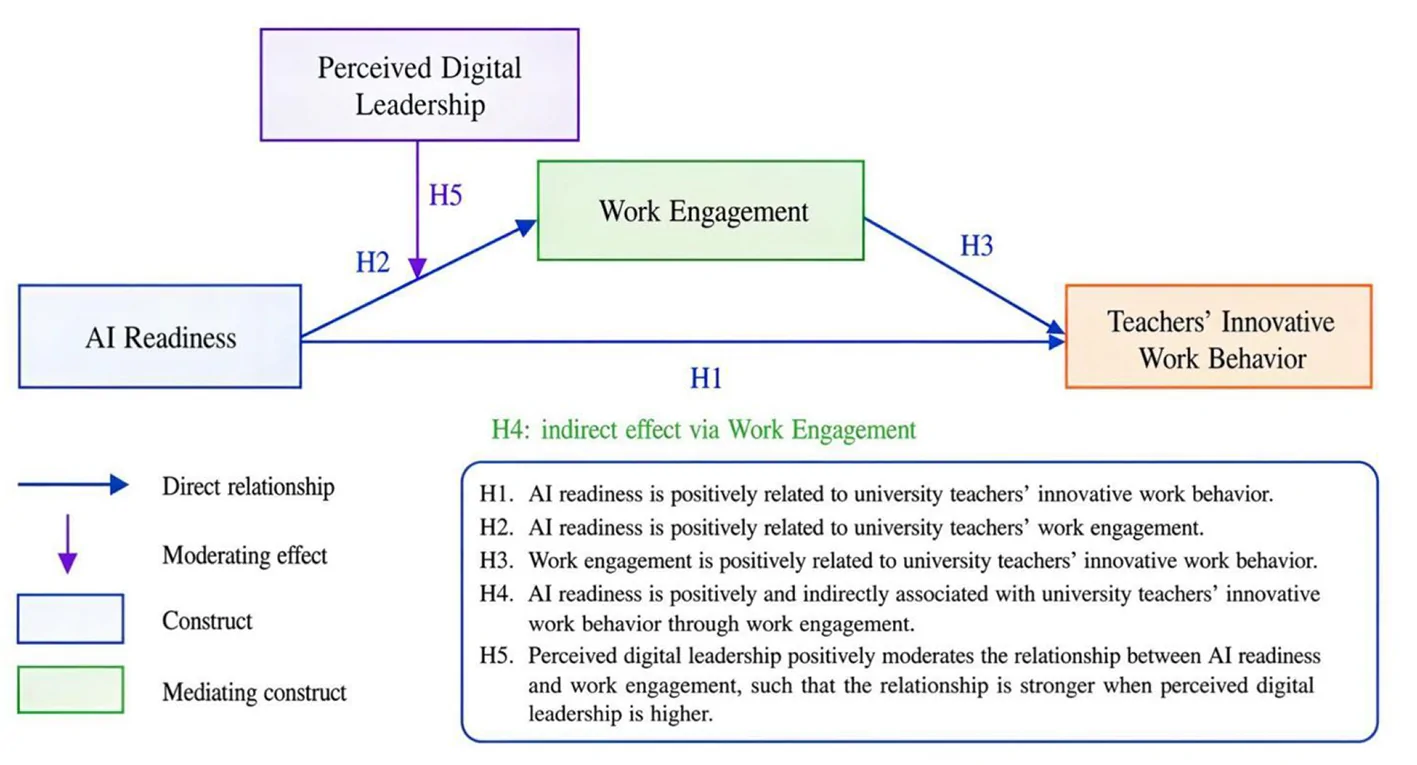
Proposed conceptual model.

Figure 1 summarizes the proposed research model. AI readiness is expected to be positively related to university teachers' innovative work behavior and work engagement, while work engagement is expected to be positively related to innovative work behavior. The model further proposes a positive indirect association between AI readiness and innovative work behavior through work engagement. Perceived digital leadership is specified as an individual-level moderator of the relationship between AI readiness and work engagement.

## 3 Methods

### 3.1 Participants and procedures

Data were collected through a cross-sectional online survey of university teachers in China. Purposive sampling was used to recruit participants who were currently employed by Chinese higher education institutions and had at least 1 year of work experience in higher education. Survey invitations were distributed online to 500 eligible teachers. Participation was voluntary. Before accessing the questionnaire, participants received information about the purpose of the study, the confidentiality of their responses, and their right to withdraw before submission. Electronic informed consent was obtained from all participants, and no personally identifying information was collected.

A total of 465 questionnaires were returned, representing an initial response rate of 93.0%. After 23 incomplete questionnaires were removed, 442 complete responses remained. A further 10 responses were excluded because they displayed zero response variance or clear straight-lining patterns. The final sample therefore comprised 432 valid responses, corresponding to 86.4% of the teachers initially contacted. All variables were measured in a single questionnaire. When completing the perceived digital leadership items, participants were instructed to evaluate the academic leader who most directly supervised or coordinated their teaching, research, or academic-service activities.

### 3.2 Measures

Established measurement scales were adapted to the context of university teachers' teaching, research, and academic-service activities. AI readiness, perceived digital leadership, and innovative work behavior were rated on five-point scales ranging from 1 (“strongly disagree”) to 5 (“strongly agree”). Work engagement was assessed using the original seven-point frequency scale of the UWES-9. Higher scores indicated higher levels of the corresponding constructs.

#### 3.2.1 AI readiness

AI readiness was measured using the 18-item instrument developed by Wang et al. (2023). The instrument comprises four dimensions: cognition (five items), ability (six items), vision (three items), and ethics (four items). The wording was adapted to reflect the use of AI in university teaching, research, and academic service (see Appendix 1). AI readiness was specified as a reflective-reflective higher-order construct, with the four dimensions modeled as reflective lower-order constructs.

#### 3.2.2 Work engagement

Work engagement was assessed using the nine-item Utrecht Work Engagement Scale (UWES-9) developed by Schaufeli et al. (2006) (see Appendix 2). The scale comprises vigor, dedication, and absorption, with three items measuring each dimension. Work engagement was modeled as a reflective-reflective higher-order construct, with vigor, dedication, and absorption serving as reflective lower-order dimensions.

#### 3.2.3 Perceived digital leadership

Perceived digital leadership was measured using an adapted follower-rated version of the six-item digital leadership scale developed by Zeike et al. (2019) (see Appendix 3). The original manager-focused wording was revised so that teachers evaluated their immediate academic leaders. A sample item is “My immediate academic leader communicates a clear vision for digital transformation.” Perceived digital leadership was modeled as a first-order reflective construct.

#### 3.2.4 Innovative work behavior

Innovative work behavior was measured using an adapted self-report version of the six-item scale developed by Scott and Bruce (1994) (see Appendix 4). The original supervisor-referent wording was rewritten in the first person to assess teachers' innovative activities in teaching, research, and academic service. Innovative work behavior was modeled as a first-order reflective construct.

### 3.3 Data analysis

IBM SPSS Statistics (Version 26) was used for data screening, descriptive statistics, and demographic analysis. SmartPLS 4 was used to estimate the measurement and structural models through partial least squares structural equation modeling. PLS-SEM was selected because the study adopted a prediction-oriented approach to theory extension and involved higher-order constructs, a statistical indirect effect, and a latent interaction.

AI readiness and work engagement were estimated using the disjoint two-stage approach, whereas perceived digital leadership and innovative work behavior were modeled as first-order reflective constructs. The measurement models were evaluated using indicator loadings, Cronbach's alpha, ρA, composite reliability, average variance extracted, and HTMT ratios. The structural model was assessed using inner VIF values, standardized path coefficients, (*R*^2^), (adjusted *R*^2^), (*f* ^2^), and predictive relevance.

Statistical significance was evaluated using 10,000 bootstrap subsamples. The statistical indirect effect of AI readiness on innovative work behavior through work engagement was used to evaluate H4. The interaction between AI readiness and perceived digital leadership was estimated using the two-stage approach. Because bootstrap tests of the conditional effects at specific levels of perceived digital leadership were not conducted, the significant interaction was illustrated descriptively using a simple-slopes plot at low (−1 SD), mean, and high (+1 SD) levels of perceived digital leadership.

## 4 Results

### 4.1 Demographic profile of respondents

Table 1 summarizes the demographic characteristics of the 432 respondents. The sample was nearly evenly divided by gender, with women accounting for 50.2% and men for 49.8%. Respondents aged 31–40 and 41–50 each represented 35.0% of the sample. Most participants held postgraduate qualifications, including doctoral degrees (43.1%) and master's degrees (38.9%). Lecturers and associate professors constituted the two largest academic-rank groups, accounting for 37.0% and 31.9%, respectively. Regarding experience in higher education, 38.0% had worked for at least 16 years, while 44.0% had between 6 and 15 years of experience.

**Table 1:** Demographic profile of respondents.

Variable	Category	Frequency (N)	Percent (%)
Gender	Male	215	49.8
	Female	217	50.2
Age	30 years or below	43	10.0
	31-40 years	151	35.0
	41-50 years	151	35.0
	51 years or above	87	20.1
Highest educational qualification	Bachelor's degree	78	18.1
	Master's degree	168	38.9
	Doctoral degree	186	43.1
Academic rank	Teaching/research assistant	30	6.9
	Lecturer	160	37.0
	Associate professor	138	31.9
	Professor	73	16.9
	No formal academic rank	31	7.2
Work experience in higher education	1–5 years	78	18.1
	6–10 years	95	22.0
	11–15 years	95	22.0
	16 years or more	164	38.0

N denotes subgroup frequency. Percentages were calculated within each demographic variable and may not total 100% because of rounding.

### 4.2 Descriptive statistics and correlations

Table 2 presents the means, standard deviations, and zero-order Pearson correlations among the main demographic and study variables. AI readiness was positively correlated with work engagement (*r* = 0.403, *p* < 0.01) and innovative work behavior (*r* = 0.443, *p* < 0.01). Work engagement was also positively correlated with innovative work behavior (*r* = 0.572, *p* < 0.01). Perceived digital leadership showed a small negative correlation with AI readiness (*r* = −0.097, *p* < 0.05), but was not significantly correlated with work engagement or innovative work behavior. These coefficients represent unadjusted bivariate associations and should not be interpreted as tests of the proposed structural relationships.

**Table 2:** Descriptive statistics and correlations.

Variables	M	SD	1	2	3	4	5	6	7	8	9
1.Gender	1.502	0.501	1.000								
2.Highest educational qualification	2.250	0.742	−0.003	1.000							
3.Work experience in higher education	2.799	1.133	0.031	−0.007	1.000						
4.Academic rank	2.803	1.033	−0.027	0.004	−0.056	1.000					
5.Age	2.653	0.911	−0.004	0.024	0.012	−0.075	1.000				
6.AR	3.471	0.678	−0.023	0.224^**^	0.032	0.071	−0.283^**^	1.000			
7.WE	4.649	1.192	−0.017	0.167^**^	0.133^**^	0.038	0.122^*^	0.403^**^	1.000		
8.PDL	3.433	0.848	0.048	−0.003	0.085	−0.032	−0.084	−0.097^*^	−0.069	1.000	
9.IWB	3.496	0.919	−0.052	0.127^**^	0.112^*^	−0.007	−0.040	0.443^**^	0.572^**^	−0.049	1.000

Note. N = 432. M = mean; SD = standard deviation. AR = AI readiness; WE = work engagement; PDL = perceived digital leadership; IWB = innovative work behavior.

^*^p < 0.05.

^**^p < 0.01 (two-tailed).

### 4.3 Assessment of potential common method variance

Because all substantive constructs were measured using self-reports collected from the same respondents in a single survey, potential common method variance could not be excluded. Harman's single-factor procedure was therefore conducted as a limited supplementary diagnostic using all 39 measurement items (Podsakoff and Organ, 1986). An unrotated principal component analysis identified nine components with eigenvalues greater than 1, which collectively explained 72.738% of the total variance. The first component had an eigenvalue of 10.552 and accounted for 27.057% of the variance. Thus, no single component accounted for the majority of the variance (see Table 3). Nevertheless, Harman's procedure has limited sensitivity and cannot establish the absence of common method bias. The result provides only supplementary evidence that a single common factor was unlikely to dominate the covariance among the measures (Podsakoff et al., 2003).

**Table 3:** Harman's single-factor test.

Component	Initial eigenvalue: total	% of variance	Cumulative %	Extraction: total	% of variance	Cumulative %
1	10.552	27.057	27.057	10.552	27.057	27.057
2	3.755	9.629	36.686	3.755	9.629	36.686
3	3.424	8.779	45.465	3.424	8.779	45.465
4	2.739	7.022	52.488	2.739	7.022	52.488
5	1.966	5.040	57.528	1.966	5.040	57.528
6	1.695	4.345	61.873	1.695	4.345	61.873
7	1.548	3.970	65.842	1.548	3.970	65.842
8	1.415	3.629	69.471	1.415	3.629	69.471
9	1.274	3.267	72.738	1.274	3.267	72.738

### 4.4 Measurement model assessment

Before evaluating the structural relationships, the reflective measurement models were assessed for indicator reliability, internal consistency reliability, convergent validity, and discriminant validity. AI readiness and work engagement were specified as reflective-reflective higher-order constructs and estimated using the disjoint two-stage approach (Sarstedt et al., 2019). In Stage 1, their lower-order dimensions were estimated using the original measurement items, together with perceived digital leadership and innovative work behavior. In Stage 2, the lower-order construct scores served as reflective indicators of AI readiness and work engagement, while perceived digital leadership and innovative work behavior retained their original reflective indicators.

The first-order measurement model results are presented in Table 4. Following Hair et al. (2019), loadings above 0.708 were preferred, reliability coefficients above 0.70 were considered satisfactory, and AVE values above 0.50 supported convergent validity. In Stage 1, the indicator loadings ranged from 0.673 to 0.943. Although some perceived digital leadership indicators were below 0.708, all remained above 0.40 and the construct retained satisfactory reliability and convergent validity. Across the Stage 1 constructs, Cronbach's alpha ranged from 0.849 to 0.936, ρA from 0.872 to 0.936, ρC from 0.876 to 0.959, and AVE from 0.544 to 0.886. As shown in Panel B, perceived digital leadership and innovative work behavior also met the criteria when reassessed in Stage 2.

**Table 4:** Assessment of the first-order reflective measurement models.

Construct	Loading ranges	Cronbach's α	ρA	ρC	AVE
Panel A. Lower-order constructs assessed in stage 1					
AR-Ability	0.789–0.852	0.907	0.908	0.928	0.682
AR-Cognition	0.819–0.852	0.893	0.894	0.921	0.701
AR-Ethics	0.876–0.891	0.908	0.908	0.935	0.783
AR-Vision	0.908–0.919	0.900	0.900	0.937	0.833
PDL	0.673–0.811	0.849	0.891	0.876	0.544
IWB	0.746–0.795	0.870	0.872	0.902	0.607
WE-Absorption	0.920–0.930	0.915	0.916	0.946	0.855
WE-Dedication	0.939–0.943	0.936	0.936	0.959	0.886
WE-Vigor	0.919–0.940	0.923	0.924	0.951	0.867
Panel B. First-order constructs reassessed in stage 2					
PDL	0.670–0.812	0.849	0.895	0.877	0.544
IWB	0.746–0.794	0.870	0.872	0.902	0.607

AR, artificial intelligence readiness; WE, work engagement; PDL, perceived digital leadership; IWB, innovative work behavior; ρA, reliability coefficient rho_A; ρC, composite reliability; AVE, average variance extracted.

The higher-order measurement results are reported in Table 5. All lower-order dimensions loaded positively and significantly on their corresponding higher-order constructs, and none of the bias-corrected bootstrap confidence intervals included zero. The loadings on AI readiness ranged from 0.697 to 0.769. Although the loadings of ability (0.697) and cognition (0.705) were slightly below the preferred threshold of 0.708, both were statistically significant and were retained to preserve the theoretically specified four-dimensional construct. AI readiness met the minimum criteria for reliability and convergent validity (Cronbach's α= 0.703, ρA = 0.706, ρC = 0.818, AVE = 0.530), although its α and ρA values were close to the recommended lower boundary. Work engagement demonstrated satisfactory reliability and convergent validity (Cronbach's α= 0.731, ρA = 0.734, ρC = 0.848, AVE = 0.650).

**Table 5:** Assessment of the higher-order reflective measurement models.

Higher-order construct	Lower-order dimension	Loading	t	p	95% CI	Cronbach's α	ρA	ρC	AVE
AI readiness	Ability	0.697	18.493	< 0.001	[0.611, 0.759]	0.703	0.706	0.818	0.530
	Cognition	0.705	20.911	< 0.001	[0.628, 0.763]				
	Ethics	0.738	25.124	< 0.001	[0.672, 0.789]				
	Vision	0.769	29.940	< 0.001	[0.710, 0.813]				
Work engagement	Absorption	0.821	43.752	< 0.001	[0.778, 0.853]	0.731	0.734	0.848	0.650
	Dedication	0.791	35.685	< 0.001	[0.739, 0.828]				
	Vigor	0.807	36.732	< 0.001	[0.757, 0.845]				

CI, bias-corrected bootstrap confidence interval.

Discriminant validity was assessed using the heterotrait-monotrait ratio of correlations. As reported in Table 6, the Stage 1 HTMT values ranged from 0.057 to 0.574, while the Stage 2 values ranged from 0.082 to 0.769. The highest value was observed between work engagement and innovative work behavior. All point estimates were below the conservative threshold of 0.850, supporting discriminant validity at the point-estimate level (Henseler et al., 2015). Comparisons between a higher-order construct and its constituent lower-order dimensions were not interpreted.

**Table 6:** Discriminant validity assessment using HTMT ratios.

Construct	AR-A	AR-C	AR-E	AR-V	PDL	IWB	WE-A	WE-D	WE-V
Panel A. HTMT ratios among the stage 1 constructs									
AR-A	—								
AR-C	0.309	—							
AR-E	0.408	0.428	—						
AR-V	0.456	0.432	0.436	—					
PDL	0.093	0.140	0.057	0.081	—				
IWB	0.351	0.389	0.392	0.424	0.082	—			
WE-A	0.309	0.294	0.313	0.369	0.059	0.574	—		
WE-D	0.264	0.242	0.276	0.260	0.061	0.526	0.493	—	
WE-V	0.227	0.272	0.203	0.247	0.107	0.554	0.531	0.518	—
Construct	AR		WE		PDL		IWB		
Panel B. HTMT ratios among the stage 2 constructs										
AR	—								
WE	0.592		—						
PDL	0.136		0.104		—				
IWB	0.605		0.769		0.082		—		

Values below the diagonal are HTMT point estimates. Comparisons between a higher-order construct and its own lower-order dimensions were not interpreted.

### 4.5 Structural model assessment

#### 4.5.1 Structural model quality

Before testing the hypothesized relationships, the structural model was assessed for collinearity, effect sizes, explanatory power, and predictive relevance (Hair et al., 2019, 2022). As reported in Table 7, inner VIF values ranged from 1.007 to 1.223, indicating that collinearity was unlikely to distort the structural estimates. AI readiness had small effects on work engagement and innovative work behavior (both *f* ^2^ = 0.097), whereas work engagement had a large effect on innovative work behavior (*f* ^2^ = 0.368). The main effect of perceived digital leadership and its interaction with AI readiness were negligible (*f* ^2^ = 0.003 and 0.015, respectively). The model explained 19.7% of the variance in work engagement and 43.4% of the variance in innovative work behavior. Both endogenous constructs had Stone-Geisser *Q*^2^ values above zero, indicating predictive relevance in the blindfolding assessment (Geisser, 1974; Stone, 1974).

**Table 7:** Structural model assessment.

Endogenous construct	Predictor/path	Inner VIF	*f* ^2^	Effect size
Panel A. Collinearity and effect sizes				
Work engagement	AR → WE	1.018	0.097	Small
Work engagement	PDL → WE	1.010	0.003	Negligible
Work engagement	AR × PDL → WE	1.007	0.015	Negligible
Innovative work behavior	AR → IWB	1.223	0.097	Small
Innovative work behavior	WE → IWB	1.223	0.368	Large
Endogenous construct	*R* ^2^	Adjusted *R*^2^	*Q* ^2^	Interpretation
Panel B. Explanatory power and predictive relevance				
Work engagement	0.197	0.191	0.123	Modest explanatory power; positive predictive relevance
Innovative work behavior	0.434	0.431	0.260	Moderate explanatory power; positive predictive relevance

#### 4.5.2 Direct and moderating effects

The bootstrapping results for the direct and moderating effects are presented in Table 8 (Hair et al., 2022). AI readiness was positively associated with innovative work behavior (β = 0.259, *t* = 6.765, *p* < 0.001, 95% bias-corrected bootstrap CI [0.183, 0.332]) and work engagement (β = 0.413, t = 10.358, *p* < 0.001, 95% bias-corrected bootstrap CI [0.329, 0.486]), supporting H1 and H2, respectively. Work engagement was also positively associated with innovative work behavior (β = 0.505, *t* = 14.559, *p* < 0.001, 95% bias-corrected bootstrap CI [0.433, 0.570]), supporting H3.

**Table 8:** Direct and moderating effects.

Hypothesis	Path	β	*t*	*p*	95% CI	*f^2^*	Decision
H1	AR → IWB	0.259	6.765	< 0.001	[0.183, 0.332]	0.097	Supported
H2	AR → WE	0.413	10.358	< 0.001	[0.329, 0.486]	0.097	Supported
H3	WE → IWB	0.505	14.559	< 0.001	[0.433, 0.570]	0.368	Supported
—	PDL → WE	−0.049	0.753	0.451	[−0.096, 0.227]	0.003	Not hypothesized (nonsignificant)
H5	AR × PDL → WE	0.110	2.050	0.040	[0.005, 0.209]	0.015	Weakly supported

The conditional main effect of perceived digital leadership on work engagement was non-significant (β= −0.049, *t* = 0.753, *p* = 0.451). This path was included as a constituent term of the moderation model and was not associated with a separate hypothesis. The interaction between AI readiness and perceived digital leadership was positive and statistically significant (β= 0.110, *t* = 2.050, *p* = 0.040, 95% bias-corrected bootstrap CI [0.005, 0.209]). This result provides weak support for H5. Although the interaction was statistically significant, its confidence interval was close to zero and its effect size was negligible (*f* ^2^ = 0.015), indicating limited practical magnitude.

**Figure 2:**
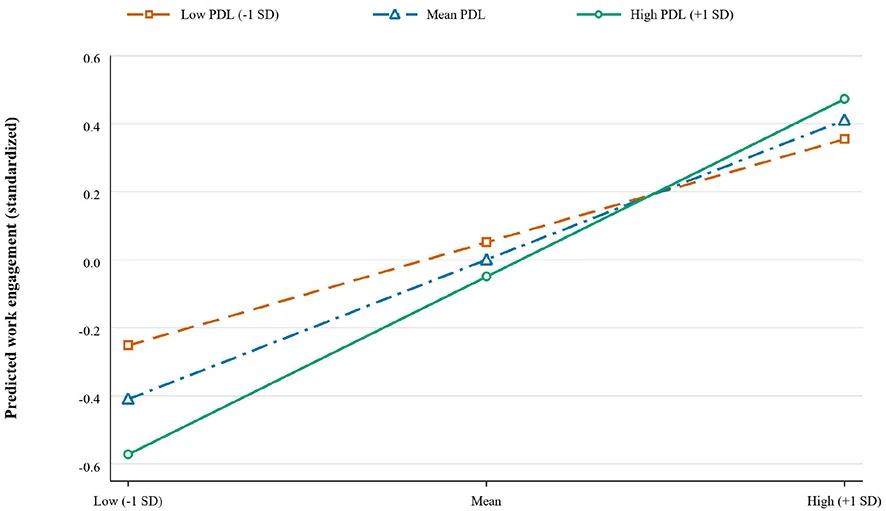
Simple-slopes plot of the moderating effect of perceived digital leadership on the association between AI readiness and work engagement.

Figure 2 presents a descriptive representation of the interaction. The positive association between AI readiness and work engagement appears steeper at higher levels of perceived digital leadership. However, because separate bootstrap tests of the conditional effects were not reported, the plotted slopes should be interpreted descriptively.

#### 4.5.3 Statistical indirect effect of work engagement

As shown in Table 9, the statistical indirect effect of AI readiness on innovative work behavior through work engagement was positive and significant (β= 0.209, *t* = 8.170, *p* < 0.001, 95% bias-corrected bootstrap CI [0.160, 0.259]), supporting H4. Because the direct association between AI readiness and innovative work behavior remained positive and significant, and the direct and indirect effects had the same sign, the observed pattern was consistent with complementary partial mediation (Zhao et al., 2010). This classification describes the statistical decomposition of the observed association and should not be interpreted as evidence of a causal mediation process because all variables were measured cross-sectionally.

**Table 9:** Indirect effect of work engagement.

Hypothesis	Indirect path	Indirect effect	*t*	*p*	95% CI	Mediation type	Decision
H4	AR → WE → IWB	0.209	8.170	< 0.001	[0.160, 0.259]	Complementary partial mediation	Supported

#### 4.5.4 Out-of-sample predictive assessment

The model's out-of-sample predictive performance was further examined using PLSpredict (Shmueli et al., 2019). As reported in Table 10, all indicator-level *Q*^2^predict values were positive, ranging from 0.089 to 0.155. For all nine target indicators, the PLS-SEM model produced slightly lower RMSE and MAE values than the linear-model benchmark. These results indicate a modest predictive advantage over the benchmark within the cross-validation procedure. Because the differences in prediction errors were small, however, they should not be interpreted as evidence of external or temporal predictive validity.

**Table 10:** PLSpredict results.

Target indicator	*Q*^2^predict	PLS-SEM RMSE	LM RMSE	PLS-SEM MAE	LM MAE
IWB1	0.109	1.120	1.128	0.921	0.924
IWB2	0.136	1.100	1.113	0.921	0.931
IWB3	0.125	1.165	1.182	0.960	0.977
IWB4	0.142	1.043	1.058	0.876	0.883
IWB5	0.146	1.131	1.143	0.939	0.942
IWB6	0.114	1.050	1.065	0.877	0.888
WE-Absorption	0.155	0.921	0.938	0.758	0.770
WE-Dedication	0.099	0.951	0.960	0.767	0.769
WE-Vigor	0.089	0.956	0.973	0.771	0.791

LM, linear-model benchmark; RMSE, root mean squared error; MAE, mean absolute error.

## 5 Discussion

Using single-wave, cross-sectional data from 432 university teachers in China, this study examined the associations among AI readiness, work engagement, perceived digital leadership, and self-reported innovative work behavior. H1–H3 were supported by significant direct associations, and H4 was supported by a significant statistical indirect effect. H5 received weak support: perceived digital leadership strengthened the association between AI readiness and work engagement, but the interaction was very small. Given the cross-sectional, single-source design, these results should be interpreted as structural associations rather than evidence of temporal or causal relationships.

AI readiness was positively associated with innovative work behavior (β = 0.259), supporting H1. This finding suggests that preparedness for AI may matter beyond the decision to adopt a particular tool. Teachers who understand AI's capabilities and limitations, can apply it to professional tasks, anticipate its implications, and exercise ethical judgment may be better positioned to recognize opportunities for improvement and develop responsible innovations in teaching, research, administration, and academic service. The result is consistent with Wang et al. (2023), who linked cognition, ability, vision, and ethics with AI-enhanced educational innovation among primary school teachers. It also accords with Held and Heubeck (2025), whose study showed that the capability to evaluate generative AI was positively associated with employees' innovative work behavior, whereas operational ability alone was insufficient. The present study extends this evidence to university teachers and to self-reported innovative work behavior that is not restricted to explicitly AI-based innovation.

The result also supports a conception of AI readiness that extends beyond technical proficiency. Effective engagement with AI requires critical evaluation of outputs, recognition of limitations, and attention to ethical and social consequences (Long and Magerko, 2020; Ng et al., 2021). A recent review of teacher education likewise identified critical, ethical, and practical capabilities as central elements of AI literacy (Sperling et al., 2024). These considerations are particularly important in higher education, where privacy, algorithmic bias, authorship, intellectual property, assessment integrity, and accountability may determine whether a novel practice is acceptable. Nevertheless, AI readiness was modeled as a reflective-reflective higher-order construct. The findings therefore concern overall readiness and do not demonstrate that cognition, ability, vision, and ethics have equally strong or independently significant associations with innovative work behavior.

AI readiness was also positively associated with work engagement (β = 0.413), supporting H2. From a JD-R perspective, AI readiness can be viewed as a technology-specific resource with resource-like functions rather than as a conventional global personal resource. Its potential value lies in helping teachers manage AI-related demands, pursue professional goals, and adapt to technological change (Xanthopoulou et al., 2007; Bakker and Demerouti, 2017). Teacher-focused evidence has linked job resources with engagement (Hakanen et al., 2006), while longitudinal research indicates that personal resources and work engagement may reinforce one another over time (Xanthopoulou et al., 2009). Related technology studies are broadly consistent with this interpretation: digital competence and digital information-literacy self-efficacy have been associated with university teachers' engagement (Sang et al., 2023; Fan et al., 2023), and technology readiness has been associated with employee engagement outside higher education (Khoza et al., 2024). These constructs are adjacent to, but not interchangeable with, AI readiness. Moreover, perceived control and reduced uncertainty were not measured in this study and remain theoretical explanations rather than demonstrated mechanisms.

Work engagement was positively associated with innovative work behavior (β = 0.505), supporting H3. Its path coefficient was numerically larger than that of AI readiness, although the difference between the two coefficients was not formally tested. The result is theoretically plausible because generating, promoting, and implementing ideas require discretionary effort, sustained attention, and persistence when innovation encounters uncertainty or resistance. Kwon and Kim's (2020) integrative review reached a similar conclusion regarding the relationship between engagement and innovative behavior. More directly, Dixit and Upadhyay (2021) found that faculty engagement was closely associated with innovative work behavior in higher education. Recent studies of academic staff and Chinese university teachers also report positive engagement-innovation relationships (Hassan et al., 2024; Zhou et al., 2025).

The statistical indirect effect of AI readiness on innovative work behavior through work engagement was positive and significant (β = 0.209), supporting H4. Because the direct and indirect associations were both positive and significant, the pattern was consistent with complementary partial mediation (Zhao et al., 2010). Conceptually, AI readiness may provide task-relevant capacity, while engagement may represent the motivational investment associated with sustained innovative activity. This interpretation is consistent with JD-R research in higher education in which engagement statistically connected work resources or conditions with innovative behavior (Dixit and Upadhyay, 2021; Hassan et al., 2024; Zhou et al., 2025). The present analysis, however, cannot establish that readiness preceded engagement or that engagement subsequently produced innovation because all variables were measured concurrently. H4 should therefore be understood as a statistical indirect association, not a verified causal mediation process.

Perceived digital leadership positively moderated the association between AI readiness and work engagement (β = 0.110, *p* = 0.040), providing weak support for H5. The plotted relationship became steeper as perceived digital leadership increased, but the available output does not show whether the individual conditional slopes were independently significant. More importantly, the interaction effect was very small (*f* ^2^ = 0.015), and its confidence interval only narrowly excluded zero. The finding is therefore consistent with, but provides limited evidence for, resource complementarity. AI readiness represents teachers' capacity to engage with AI, whereas perceived digital leadership may signal that using this capacity is encouraged and aligned with unit priorities. (Tummers and Bakker, 2021) review similarly suggests that leadership can shape the availability and use of job and personal resources. Related studies have connected digital leadership with teachers' technology integration (Alajmi, 2022) and employees' innovative work behavior (Ahmed et al., 2024), but neither study tested the specific AI-readiness-by-leadership interaction examined here.

The nonsignificant conditional coefficient for perceived digital leadership should not be interpreted as showing that leadership is unrelated to engagement under all conditions. In a model containing an interaction term, the coefficient represents the association between perceived digital leadership and work engagement when AI readiness is at its reference value. Nor do the findings demonstrate that leadership operates exclusively as a moderator. Social exchange theory offers one possible explanation because supportive treatment may encourage employees to reciprocate through stronger engagement (Saks, 2006). However, leader-member exchange, perceived obligations, and reciprocity were not measured. The findings are therefore compatible with, rather than a direct test of, this social-exchange explanation.

### 5.1 Theoretical implications

Rather than merely combining previously supported relationships, this study addresses an unresolved resource-conversion question: whether and how AI-specific preparedness is associated with innovative behavior beyond AI adoption or explicitly AI-enhanced innovation. Three theoretical insights emerge.

First, the study extends the conceptual boundary of AI readiness by examining its cross-domain relevance. AI readiness reflects teachers' cognition, ability, vision, and ethical judgment concerning AI, whereas innovative work behavior encompasses the generation, promotion, and implementation of practices across teaching, research, administration, and academic service. Their association is therefore consistent with the possibility that a technology-specific personal resource has behavioral relevance beyond its original domain. However, because AI readiness was modeled as an overall higher-order construct, the study cannot determine whether its four dimensions contribute equally to innovation.

Second, the study distinguishes resource possession from motivational activation. AI readiness represents teachers' AI-related capacity, work engagement reflects the energy and involvement invested in mobilizing that capacity, and innovative work behavior represents its potential behavioral expression. The statistical indirect association through work engagement is consistent with a capacity–motivation–behavior process. This refines the JD-R motivational perspective by applying it to the conversion of an emerging domain-specific resource, although the cross-sectional design cannot establish temporal or causal mediation. Because no explicit AI-related job demands were included, these findings provide evidence only for a resource-focused interpretation of the JD-R motivational process. They do not test the health-impairment process, demand-resource interactions, or the complete JD-R dual-process framework.

Third, the moderation result provides weak and tentative evidence of resource complementarity across different levels of content specificity. AI readiness is an AI-specific personal resource, whereas perceived digital leadership represents a broader contextual resource concerning digital transformation. AI and digitalization are therefore related but not equivalent concepts. The positive interaction is consistent with the possibility that general digital direction, legitimacy, and encouragement help teachers mobilize their AI-related capacities. However, the interaction effect was negligible (*f* ^2^ = 0.015), and its confidence interval was close to zero. PDL should therefore not be interpreted as a strong boundary condition or as a substitute for AI-specific leadership. The limited effect may be consistent with only partial content congruence between a broad contextual resource and a highly specific personal resource. This result raises a more precise theoretical question for future research: whether AI-specific leadership, strategy, support, and governance would more strongly activate teachers' AI readiness than general digital leadership. Because the present study did not measure AI-specific leadership, this interpretation remains preliminary.

### 5.2 Practical implications

The findings suggest that universities may benefit from assessing and developing faculty AI readiness rather than relying exclusively on generic tool demonstrations. Development initiatives could address understanding AI's functions and limitations, applying it to academic tasks, anticipating changes in professional roles, and making responsible decisions concerning privacy, bias, intellectual property, transparency, and accountability. Because the structural analysis examined overall AI readiness, it does not establish that any one dimension should receive greater priority than the others.

Capability development should be accompanied by working conditions that support engagement. Universities may consider practice-based training, discipline-specific cases, technical consultation, protected time for experimentation, small pilot grants, interdisciplinary learning communities, and recognition of responsible innovation. These recommendations are consistent with the observed association between work engagement and innovative work behavior, but they should be regarded as evidence-informed proposals rather than interventions whose effectiveness was demonstrated by the present study.

Immediate academic leaders may support this process by connecting AI use with departmental priorities, directing teachers to relevant expertise, and responding consistently to ethical concerns. Nevertheless, the very small interaction effect cautions against relying on leadership communication alone. Leadership should complement, rather than replace, professional development, suitable infrastructure, manageable workloads, access to approved tools, and institutional governance. Consistent with UNESCO's guidance, universities should combine capacity building with human-centered governance, privacy protection, and ethical validation.

### 5.3 Limitations and future research

First, all constructs were measured through a single-wave, cross-sectional survey completed by the same respondents. Harman's single-factor procedure cannot eliminate common method bias, and the design does not establish temporal order, reciprocity, or causality. Moreover, self-reported innovative work behavior may reflect perceived rather than implemented innovation and may be affected by socially desirable responding. Future research should employ longitudinal, cross-lagged, or intervention-based designs and combine self-reports with supervisor evaluations and behavioral indicators of implemented innovation.

Second, purposive sampling and limited information about the sampling frame, institutional distribution, disciplines, and geographical coverage restrict generalizability. Teachers may also have been nested within academic units and universities, although the analysis treated observations as independent. In addition, institutional AI access, disciplinary norms, prior AI experience, workload, and innovation climate were not modeled. Future studies should use more diverse samples, multilevel designs, and theoretically justified control variables.

Third, the model did not include explicit AI-related job demands. It therefore cannot determine whether learning burden, technostress, professional uncertainty, or ethical strain suppresses or conditions the positive association between AI readiness and work engagement. Future research should examine AI-related resources and demands simultaneously and test their mediating, suppressing, moderating, or nonlinear effects. Dimension-level analyses may also reveal whether the four dimensions of AI readiness have different motivational and strain-related consequences.

Fourth, the moderator concerned digital leadership broadly rather than leadership specifically directed toward AI. The very small interaction effect may partly reflect this limited content correspondence, although the present data cannot test that explanation. Future research should develop or adopt validated measures of AI-specific leadership and compare their incremental and moderating effects with those of general digital leadership.

Finally, the measurement specifications require further examination. The higher-order AI-readiness construct showed reliability estimates close to the recommended lower boundary, several PDL indicators had relatively low loadings, and the high reliability of some work-engagement dimensions may indicate item similarity. The AI-readiness scale was also adapted from school teaching to university work. Future studies should compare alternative higher-order specifications, test dimension-level relationships and measurement invariance, and preregister replications of the small AI readiness × PDL interaction using larger samples, conditional-effect tests, bootstrap confidence intervals, and regions of significance.

### 5.4 Conclusion

This study found that AI readiness was positively associated with university teachers' work engagement and innovative work behavior, while work engagement was also positively associated with innovative work behavior. Taken together, the findings are consistent with a resource-conversion account in which an AI-specific personal resource may have cross-domain relevance for broader innovative behavior, with work engagement representing a plausible form of motivational activation. However, the cross-sectional design does not establish the temporal or causal process implied by this account. The interaction between AI readiness and perceived digital leadership was positive but negligible in magnitude, providing only weak support for PDL as a boundary condition of the AI readiness-engagement association. Because PDL captures leadership concerning digital transformation broadly rather than AI-specific leadership, this finding should be interpreted cautiously and should not be taken as evidence that general digital leadership is a strong or theoretically equivalent substitute for AI-specific leadership. Overall, universities seeking to support responsible innovation should combine faculty AI-readiness development with conditions that sustain engagement, appropriate institutional support, and clear AI governance.

## Data Availability

The original contributions presented in the study are included in the article/Supplementary material, further inquiries can be directed to the corresponding authors.
