# Supplementary material for: Artificial intelligence readiness and innovative work behavior among Chinese university teachers: the indirect role of work engagement and the moderating role of perceived digital leadership
Source: Front Psychol. 2026 Sep 9;17:1958619. doi: 10.3389/fpsyg.2026.1958619 (PMC13597695; doi:10.3389/fpsyg.2026.1958619)
Supplement: Supplementary file 1 [file Data_Sheet_1.pdf]

Appendix 1

AI READINESS: AI Readiness Scale (ARS) (Wang et al.2023). Cronbach's α : Cognition =.93; Ability = .97; Vision = .90; Ethics = .93

Instructions: Please answer the following questions about yourself by indicating the extent of your agreement using the following Likert scale.

Strongly Disagree	Disagree	Neither agree nor disagree	Agree	Strongly Agree
1	2	3	4	5

No.AR	Items	Likert Scale				
AR-C1	I understand the basic concepts and principles underlying artificial intelligence.	1	2	3	4	5
AR-C2	I am familiar with the main functions of AI tools relevant to university work.	1	2	3	4	5
AR-C3	I can distinguish between tasks for which AI is suitable and those for which it is not suitable.	1	2	3	4	5
AR-C4	I understand the strengths and limitations of AI-generated outputs.	1	2	3	4	5
AR-C5	I understand how AI may influence university teaching, research, and academic service.	1	2	3	4	5
AR-A1	I can select appropriate AI tools for different professional tasks.	1	2	3	4	5
AR-A2	I can provide clear instructions or prompts to obtain useful results from AI tools.	1	2	3	4	5
AR-A3	I can integrate AI tools into my teaching, research, or academic service activities.	1	2	3	4	5
AR-A4	I can evaluate the accuracy and relevance of AI-generated information.	1	2	3	4	5

AR-A5	I can revise AI-generated outputs according to my professional judgment.	1	2	3	4	5
AR-A6	I can independently learn how to use unfamiliar AI tools or seek appropriate support when necessary.	1	2	3	4	5
AR-V1	I can anticipate how AI may change university work in the future.	1	2	3	4	5
AR-V2	I can identify opportunities to use AI to improve teaching, research, and academic service.	1	2	3	4	5
AR-V3	I can develop realistic plans for the responsible integration of AI into my professional work.	1	2	3	4	5
AR-E1	I consider privacy and confidentiality when using AI tools.	1	2	3	4	5
AR-E2	I consider possible bias and unfairness in AI-generated outputs.	1	2	3	4	5
AR-E3	I disclose or acknowledge my use of AI when required by academic-integrity standards.	1	2	3	4	5
AR-E4	I take responsibility for my use of AI and follow relevant laws, policies, and institutional regulations.	1	2	3	4	5

Appendix 2

WORK ENGAGEMENT: Utrecht Work Engagement Scale–9（UWES-9）（Schaufeli, Bakker, and Salanova 2006. Cronbach’s α =.85－.92

Instructions: Please indicate how often you experience each of the following feelings in relation to your work using the following frequency scale.

**Never**	**Almost never**	**Rarely**	**Sometimes**	**Often**	**Very often**	**Always**
**1**	**2**	**3**	**4**	**5**	**6**	**7**

No.WE	Items	Likert Scale						
WE-V1	I feel energetic when carrying out my university work.	1	2	3	4	5	6	7
WE-V2	I remain mentally resilient when my work becomes difficult.	1	2	3	4	5	6	7
WE-V3	I am willing to invest substantial effort in my work.	1	2	3	4	5	6	7
WE-D1	I regard my university work as meaningful and important.	1	2	3	4	5	6	7
WE-D2	I feel enthusiastic about my work.	1	2	3	4	5	6	7
WE-D3	My work inspires me to perform at my best.	1	2	3	4	5	6	7
WE-A1	I become deeply concentrated when performing my work.	1	2	3	4	5	6	7
WE-A2	I am often fully immersed in my work activities.	1	2	3	4	5	6	7
WE-A3	It is sometimes difficult for me to disengage from my work because I am deeply involved in it.	1	2	3	4	5	6	7

Appendix 3

PERCEIVED DIGITAL LEADERSHIP: Digital Leadership Scale) (DLS) (Zeike et al.2019). Cronbach’s α =.87

Instructions: Please answer the following questions about yourself by indicating the extent of your agreement using the following Likert scale.

Strongly Disagree	Disagree	Neither agree nor disagree	Agree	Strongly Agree
**1**	**2**	**3**	**4**	**5**

No.DL	Items	Likert Scale				
DL1	My immediate academic leader uses digital technologies competently in their work.	1	2	3	4	5

DL2	My immediate academic leader understands the opportunities and risks associated with digital technologies.	1	2	3	4	5
DL3	My immediate academic leader serves as a positive example in learning and using new digital technologies.	1	2	3	4	5
DL4	My immediate academic leader communicates a clear vision for digital transformation.	1	2	3	4	5
DL5	My immediate academic leader encourages teachers to experiment with new digital approaches.	1	2	3	4	5
DL6	My immediate academic leader provides support and coordination to advance digital transformation.	1	2	3	4	5

Appendix 4

INNOVATIVE WORK BEHAVIOR: Innovative Behavior Scale(IBS) (Scott and Bruce 1994).Cronbach's α =.89

Instructions: Please answer the following questions about yourself by indicating the extent of your agreement using the following Likert scale.

Strongly Disagree	Disagree	Neither agree nor disagree	Agree	Strongly Agree
**1**	**2**	**3**	**4**	**5**

No.IWB	Items	Likert Scale				
IWB1	I identify opportunities to improve existing teaching, research, or academic-service practices.	1	2	3	4	5
IWB2	I search for new methods, technologies, or approaches that could improve my work.	1	2	3	4	5
IWB3	I generate original ideas or solutions to work-related problems.	1	2	3	4	5
IWB4	I communicate and promote promising ideas to obtain support from others.	1	2	3	4	5
IWB5	I develop practical plans for implementing new ideas.	1	2	3	4	5
IWB6	I test, refine, and apply new ideas in my teaching, research, or academic-service activities.	1	2	3	4	5
